# Generation of Human 3D Airway Assembloids for Advanced Modeling

**DOI:** 10.7150/ijbs.113920

**Published:** 2025-10-01

**Authors:** Maria Chiara Iachini, Alberto Coglot, Dorian Tace, Noemi Elia, Francesco Rusconi, Federica Cosentino, Gianluca Lopez, Mariacristina Crosti, Tuğba Dursun Usal, Edoardo Scarpa, Antonio D'Amore, Vitale Miceli, Lorenzo Rosso, Lorenza Lazzari

**Affiliations:** 1 Unit of Cell and Gene Therapies, Fondazione IRCCS Ca' Granda Ospedale Maggiore Policlinico, Milan, Italy.; 2 Ri.MED Foundation, Palermo, Italy.; 3 Division of Pathology, Fondazione IRCCS Ca' Granda Ospedale Maggiore Policlinico, Milan, Italy.; 4 Department of Biomedical, Surgical and Dental Sciences, University of Milan, Italy.; 5 National Institute of Molecular Genetics “Romeo and Enrica Invernizzi” (INGM), Milan, Italy.; 6 Department of Pharmaceutical Sciences (DISFARM), University of Milan, Italy.; 7 University of Pittsburgh, Departments of Surgery and Bioengineering McGowan Institute for Regenerative Medicine, Pittsburgh, USA.; 8 Research Department, IRCCS ISMETT (Istituto Mediterraneo per i Trapianti e Terapie ad alta Specializzazione), Palermo, Italy.; 9 Thoracic Surgery and Transplantation Unit, Fondazione IRCCS Ca' Granda Ospedale Maggiore Policlinico, Milan, Italy.; 10 Department of Pathophysiology and Transplantation, University of Milan, Italy.

**Keywords:** lung, airway, 3D model, organoids, assembloids

## Abstract

The development of physiologically relevant in vitro 3D models is crucial for studying lung biology and disease mechanisms. While airway organoids have significantly improved our ability to mimic lung tissue, they lack key nonepithelial components that are essential for tissue homeostasis. Here, we describe the generation of human airway assembloids, combining airway organoids, stromal fibroblasts, and endothelial cells to better replicate the native lung environment. The model was generated from healthy lung tissue donors by using a scaffold-free culture system to promote cell self-organization. Assembloids exhibited long-term viability, maintained typical airway epithelial markers, and demonstrated functional characteristics, such as mucus production and ciliary beating. This technology provides a powerful platform for studying airway physiology, disease mechanisms, and therapeutic approaches, with potential applications in regenerative and personalized medicine. Our study established a novel, reproducible 3D assembloid model of the human airways, bridging the gap between traditional organoid cultures and complex tissue engineering strategies.

## Introduction

Organ functionality is dependent on synergistic interactions between multiple cell types within a three-dimensional (3D) structure 1. Most *in vitro* cell culture techniques still rely on two-dimensional (2D) monolayers although the transfer of cells from their native environments to 2D cultures leads to losses of tissue-specific cellular functions and architectural cues 2,3. Consequently, resembling a 3D architecture is essential for developing more accurate *in vitro* model systems 4,5.

In the last decade, our understanding of human stem cell biology has grown and the availability of 3D cell culture technologies has increased significantly, enabling the development of stem cell-derived organoid cultures that mimic organ structure and function. Organoids are typically derived from either pluripotent or tissue-specific adult stem cells (e.g., from the brain 6, liver 7, pancreas 8,9, and lung 10,11) that are cultured *in vitro* under specific conditions to promote self-organization similar to the architectures of the organs they represent. Therefore, organoids are innovative 3D models that recapitulate key features of organ physiology, including intercellular interactions, tissue organization and functionality, thus facilitating studies of organ development and pathogenesis, drug discovery and regenerative medicine 12-16.

3D cell culture systems provide significant advantages over traditional 2D cultures because they replicate native tissue architecture more reliably. Although organoids represent a major advance in the 3D culture field, they remain simplified models of natural tissues. Notably, they lack key nonepithelial components, and particularly fail to recapitulate the cell-cell interactions of the natural organ. Functional networks between epithelia and surrounding tissues are essential in the regulation of development and in organ-specific pathogenesis. Replication of the natural microenvironment that includes fibroblasts and endothelial cells is expected to generate high-fidelity models that better mimic the natural organ.

Assembloids have emerged as next-generation 3D tissue models that integrate multiple cell types and offer a higher fidelity representation of organ complexity and function. First introduced by Pasca 17, the term “assembloid” refers to a 3D multicellular model that combines multiple organoids or specialized cell types. These advanced models structurally mimic key physiological and pathological processes such as cell migration and intercellular signaling.

Assembloids that display liver, kidney, heart, and brain tissues are available 18. However, to our knowledge, no assembloids of healthy pulmonary tissue have been created, although lung cancer assembloids have already been described 19. Herein, we report the development of a functional assembloid that recapitulates the healthy airways by combining an airway organoid with a stromal fibroblast compartment, isolated from the same tissues, and a human endothelial cell line. This cellular composition enabled a more accurate representation of the native airway microenvironment that includes intercellular interactions, essential to organ homeostasis.

## Materials & Methods

### Sample collection

Healthy human lung tissue was obtained from the Thoracic Surgery and Lung Transplant Unit, Fondazione IRCCS Ca' Granda Ospedale Maggiore Policlinico, Milan, Italy. The use of human specimens was approved by the Ethical Committee of Fondazione IRCCS Ca' Granda Ospedale Maggiore Policlinico Milano (CE-0006851). Young donors underwent minimal invasive wedge lung resection indicated for spontaneous pneumothorax.

### Human airway organoid generation and culture

Human airway organoids (hAO) were generated from tissue-resident adult stem cells (Fig. Supplementary 1A) as previously described 11. After 10-21 days of culture, hAO were removed from the Cultrex-UltiMatrix RGF-BME (BME001-05, Bio-Techne) in cold Dulbecco's phosphate buffered saline without Ca^2+^and Mg^2+^(DPBS, ECB4004L Euro Clone) and enzymatically dissociated with trypsin-EDTA solution (10X, T4174 Sigma-Aldrich) for 5 min. Cells were then counted in a Burker chamber and seeded in Cultrex-UltiMatrix RGF-BME (ratio 1:6-1:12), enabling the formation of new organoids. Unless specified, hAO were analyzed after at least 15-20 days post-seeding at the indicated passage. hAO were characterized by RT-qPCR, flow cytometry, immunohistochemistry, and immunofluorescence staining at different passages. All cell cultures were routinely tested for mycoplasma contamination by MycoAlert Assay Control Set (LT07-518, Lonza).

### Lung fibroblast isolation and culture

Lung fibroblasts (LF) were isolated from the same lung biopsy specimens. Once minced, one or two pieces were seeded on 6-well culture plate with high-glucose Dulbecco's Modified Eagle Medium (DMEM, ECM0102L, Euro Clone) supplemented with 1% GlutaMAX (35050-038, Gibco), 10% fetal bovine serum (FBS, 10270-106, Gibco), and 1% antibiotic-antimycotic solution (ECM0010D, Euro Clone). After 6-8 days, fibroblasts began migrating from the tissue fragments and adhered to the plastic surface, establishing a classical stromal monolayer (Fig. Supplementary 1B). LF were routinely grown on DMEM 10%FBS, 1%GlutaMAX, 1% penicillin-streptomycin (P4333, Sigma-Aldrich) at 37°C in a humidified atmosphere of 5% CO_2_ and passaged with 0,05% trypsin (25-054-CI, Corning) and 0,02% EDTA (E7889, Sigma-Aldrich). RT-qPCR, flow cytometry, and immunofluorescence were used to confirm their identity. Cultured fibroblasts (80% confluence) were used for experiments from passages 3 to 6.

### RT-qPCR analysis

Total RNA was isolated from at least five independent hAO using TRIzol reagent (FS-881, Fisher Molecular Reagent) according to the manufacturer's instructions. RNA concentration was measured using a NanoDrop ND-100 spectrophotometer (NanoDrop Technologies). To perform an RT-qPCR assay, cDNA was synthesized from 500ng of total RNA with SuperScript IV VILO Master Mix with ezDNase (11766050, Invitrogen). The cDNA was diluted 10-fold, and 1μL of the sample was used as a template for RT-qPCR analysis with PowerUp SYBR Green Master Mix (A25742, Applied Biosystems) on a QuantStudio3 Real-Time PCR Instrument (A28131, Applied Biosystems). Relative expression levels of the specific target genes (Table 1) 20,21 were determined using the 2^-ΔCt method and normalized to the geometric mean of the GAPDH and HPRT mRNA levels.

### Flow cytometry

hAO were dissociated with trypsin-EDTA solution 1X in DPBS for 5 min to obtain a single-cell suspension. A minimum of 100,000 cells per tube were stained for each sample. For surface marker analysis, cells were incubated with fluorophore-conjugated antibodies in a total volume of 100μL of DPBS for 10 min in the dark at room temperature (RT), washed with 1ml of DPBS, and resuspended in 200μL of PBS. For intracellular marker analysis, cells were fixed in 500μL of 1% paraformaldehyde (PFA,15710, ChemCruz) for 15 min at RT, washed with 2ml of DPBS, permeabilized and incubated with 500μL of 0.05% saponin (SAE0073-10G, Sigma-Aldrich), 2% FBS, and the primary antibody. Next, permeabilized and stained cells were washed with 1ml of 0.05% saponin, 2% FBS, and incubated with the secondary antibody for 10 min at RT. After two additional washing steps, cells were resuspended in 200μL of DPBS.

LF were collected, washed once with DPBS, resuspended in 100µl staining solution (DPBS, 1% BSA containing specific antibodies), and kept for 20 min in the dark at 4 °C. Cells were then washed and resuspended in 200µl of DPBS 1% BSA solution. Flow cytometry of LF was completed using the same protocol described above for hAO. Monoclonal antibodies are reported in Table 2.

Stained cell suspensions of hAO and LF were analyzed with the FACSCanto II cytometer with FACSDiva analysis software (Becton Dickinson, BD) or with the BD FACS Lyric (BD) using FlowJo software (BD). Acquired events were plotted against forwarding scatter-height and forward scatter-area (FSC-A) to exclude cell doublets (P1 gate) and then against FSC-A and side scatter-area physical parameters to select live cell populations excluding debris (P2 gate). At least 10,000 P2 events were acquired. Markers of interest were measured in histograms or dot plots by an analytical gate for marker-positive events. Histogram data were presented as mean *±* SEM of at least n=3 independent experiments.

### MTT assay

hAO were washed twice with DPBS without Ca^2+^ and Mg^2+^ and 0.5 mg/mL of thiazolyl blue tetrazolium bromide (M5655; Sigma-Aldrich) dissolved in DMEM without phenol red (D1145; Sigma-Aldrich) (MTT Solution) was added to each well. hAO were then incubated in the dark at 37 °C with 5% CO_2_ in a humidified atmosphere for 2 h. Next, the MTT solution was removed and 96% ethanol (528,151; Carlo Erba) was added to each well. Plates were next placed on a rocking shaker at 37 °C with 5% CO_2_ in a humidified atmosphere for at least 2 h. Optical density was measured at 570 nm on a microplate reader (TECAN Infinite 200 PRO).

### Immunohistochemistry and immunofluorescence staining

hAO were washed with DPBS and fixed for 15-30 min in cold 4% PFA, followed by dehydration, paraffin embedding, sectioning (3µm thickness), and standard hematoxylin and eosin (H&E), and Alcian-blue periodic acid Schiff (AB-PAS) staining protocols. Images were acquired using the Leica Aperio AT2 (objectives 20X and 40X).

Unstained 3µm sections were deparaffinized and incubated overnight at 4°C with primary antibodies. On the following day, samples were washed and incubated with secondary antibodies and DAPI (1μg/ml in DPBS, ThermoFisher Scientific). ProLong Gold Antifade Reagent (P36930, ThermoFisher Scientific) was used as a mounting medium. Images were obtained on a confocal microscope (Leica SP8, 20X objective).

To conduct immunofluorescence staining of hAO, samples were removed from the extracellular matrix (ECM), washed with DPBS, fixed with 4% PFA for 40 min on ice, permeabilized with 0.3% Triton X-100 (9036-19-5, Sigma-Aldrich) in DPBS for 40 min at RT, washed again with DPBS, and incubated three times for 10 min with 100mM glycine (1610724, Bio-Rad) in DPBS at RT. After another wash in DPBS, they were blocked for 12h at RT with blocking solution containing 0.1% BSA (11922.03, Serva), 0.2% Triton X-100, 0.05% Tween-20 (P7949, Sigma Aldrich), and 10% goat serum (S26, ThermoFisher Scientific) in DPBS.

Samples were incubated with primary antibodies diluted in blocking solution for 24h at 37 °C in a shaker, then washed carefully with DPBS and incubated with secondary antibodies and DAPI for 2 h at 27 °C. Samples were washed briefly and transferred into a µ-Slide 18 well Glass Bottom (81817, Ibidi GmbH) with ProLong Glass Antifade Mountant (P36984, ThermoFisher Scientific) before imaging with a confocal microscope (Leica SP8, 20X objective). Primary and secondary antibodies are listed in Table 2.

### Multi-photon microscopy analysis

Imaging was conducted by using a multi-photon (MTP) Leica SD 8 microscope configured with 750nm excitation, 3-5% laser power, and a sampling rate of 40μs/pixel. Two emission signals with broad spectral ranges were utilized to effectively capture autofluorescent signals. Emission signals were collected in the ranges of 404-506nm and 526-627nm. Furthermore, sampling rates were fine-tuned to enhance image quality. The broad spectral range of the emission signals enabled effective capture of autofluorescent signals, eliminating the need for external dyes. Three timepoints (days 4, 12, and 16) were selected to observe changes in hAO morphology.

### Lentivirus production and infection

Human umbilical vein endothelial cells (HUVEC/TERT2) (Evercyte GmgH, Wien - CHT-006-0008) were grown in culture flasks coated with 0.1% gelatin with an endothelial cell growth medium BulletKit (CC-3124, Lonza) supplemented with 10% FBS and 20 µg/ml G418 (ant-gn-5, InvivoGen) at 37°C in a humidified atmosphere of 5% CO_2_.

Lentivirus (LV) was generated by transfecting a HEK-293T cell line (ATCC CRL-3216™) with packaging plasmids (pMDLg/pRRE Addgene plasmid #12251; pRSV-Rev Addgene plasmid #12253; pMD2.G Addgene plasmid #12259) and transfer plasmids (pRRLsin.cPPT.PGK-GFP.WPRE Addgene plasmid #12252) complexed with Polyethylenimine Max (49553-93-7, Kyfora Bio Polysciences). Medium was changed 6h after transfection. Supernatants were collected 72h after transfection, filtered through a 0.33μm PVDF filter (SLGV033RS, Millipore), and then ultracentrifuged for 2h at 70,000 *g* at 4 °C. The LV pellet was then resuspended in DPBS at 4 °C and stored in aliquots at -80 °C. The concentrated LV were titrated on HEK-293T. The percentages of GFP^+^ positive cells were evaluated by flow cytometry 5 days post-transduction, and LV titers were calculated and expressed in transducing unit (TU) ml^-1^. HUVEC/TERT2 cells were infected with the supernatants at a multiplicity of infection of 5-6, and polybrene (TR-1003, Sigma Aldrich) was added to a final concentration of 8 µg/ml. They were successfully infected with the eGFP-expressing lentiviral vector as demonstrated by confocal microscopy and flow cytometry (Fig. Supplementary 2A). To confirm HUVEC/TERT2 cell line identity as internationally requested, short tandem repeat analysis was performed as previously described (Fig. Supplementary 3A, 3B) 22.

### Assembloid generation

Cell ratios and culture conditions were optimized to achieve assembloid formation and growth. Before establishing the highest-yielding culture conditions, the viabilities of single cell populations (i.e., LF, hAO, and GFP^+^-HUVEC/TERT2 cells) in defined final assembloid media were tested (data not shown). In addition, different substrates and culture supports were evaluated. Finally, hAO (p4-6), LF (p4-6) and GFP^+^-HUVEC/TERT2 cells were counted separately, then centrifuged together at 1400 rpm for 5 min, and seeded in 1:1:1 ratio in BIOFLOAT 96-well plates (F202003, faCellitate) without Cultrex UltiMatrix RGF-BME (Fig. Supplementary 2B). The final medium was composed of hAO and HUVEC/TERT2 media in a 1:1 ratio with 1% FBS (final concentration), and supplemented with 10 µM Rock Inhibitor for the first 48 h. The medium was changed every two days.

Due to the novelty of the culture process, the formation of human airway 3D structures was observed daily and monitored under light microscopy. The first step was the identification of formed compact aggregates facilitating the selection of the best culture techniques. Formed multicellular clusters were subjected to immunofluorescence, immunohistochemistry (H&E and AB-PAS) and scanning electron microscopy (SEM) to identify assembloids. Crucial parameters such as viability, mucus formation, and ciliary beating were investigated at days 8, 16, and 24.

### Assembloid viability

Because the viability of a 3D model is crucial to ensure the maintenance of tissue-like functionality and cellular interactions and to thereby enable the accurate modeling of biological processes and responses, we evaluated this important parameter until day 40. Cell viability was assessed using the 3D organoid cell viability assay (AKES081, Assay Genie) following the manufacturers' instructions. This kit relies on the enzymatic reduction of WST-8 tetrazolium salt by mitochondrial dehydrogenases, generating an orange-colored product whose intensity correlates with the number of viable cells, as measured at 450 nm. The pre-assay incubation time ranged from 1 to 4 h. The assay was performed at 8, 16, and 40 days to monitor assembloid viability.

### Scanning electron microscopy

SEM was conducted on assembloids that were fixed with 2.5% glutaraldehyde in PBS overnight. Samples were rinsed with PBS and milliQ water for 5 min and then dehydrated in ethanol solutions of 30 - 50 - 70 - 90 -100% in H_2_O for five min twice. Hexamethyldisilazane (HMDS) drying was completed by immersing samples in 100% HMDS for 10 min followed by mounting on SEM stubs. On the following day, the samples were sputter-coated (Quorum Tech) and scanned by SEM EVO 10 (Zeiss).

### Forskolin-induced swelling assay

To assess the functionality of airway epithelial cells, activation of the cystic fibrosis transmembrane conductance regulator (CFTR) chloride channel was evaluated using the Forskolin-induced swelling (FIS) assay. hAO at different passages and airway assembloids after 16 days from seeding were treated with 20μM Forskolin (#1099, Tocris), freshly diluted in culture medium. hAO were exposed to Forskolin for 72 hours, whereas assembloids were treated for up to 8 days. Swelling of the organoids and assembloids was monitored over time as an indirect measure of CFTR activity. Images were acquired at regular intervals, and changes in size were quantified using Fiji software (National Institute of Health, US).

### Organoid and assembloid size measurement

To determine the size of hAO and assembloids, bright-field images were acquired with a Nikon Eclipse TS100 microscope equipped with a DS-Fi1 Nikon camera. The number, the diameter and the area of organoids and assembloids were measured using Fiji Software (National Institute of Health, US) by two blinded operators. At least three independent experiments were analyzed.

### Statistical analysis

All the quantitative data are presented as mean ± SEM, as indicated in each figure legend with the specific statistical test used. All experiments were repeated at least three times; each performed on samples from different donors. All statistical tests were performed using GraphPad Prism 9 and statistical significance (*p-value*) in figure asterisks mean *p<0.05, **p<0.01, ***p<0.001. P values <0.05 were considered significant, while p values >0.05 were not reported.

## Results

### Generation and characterization of human airway organoids

**Figure 1:**
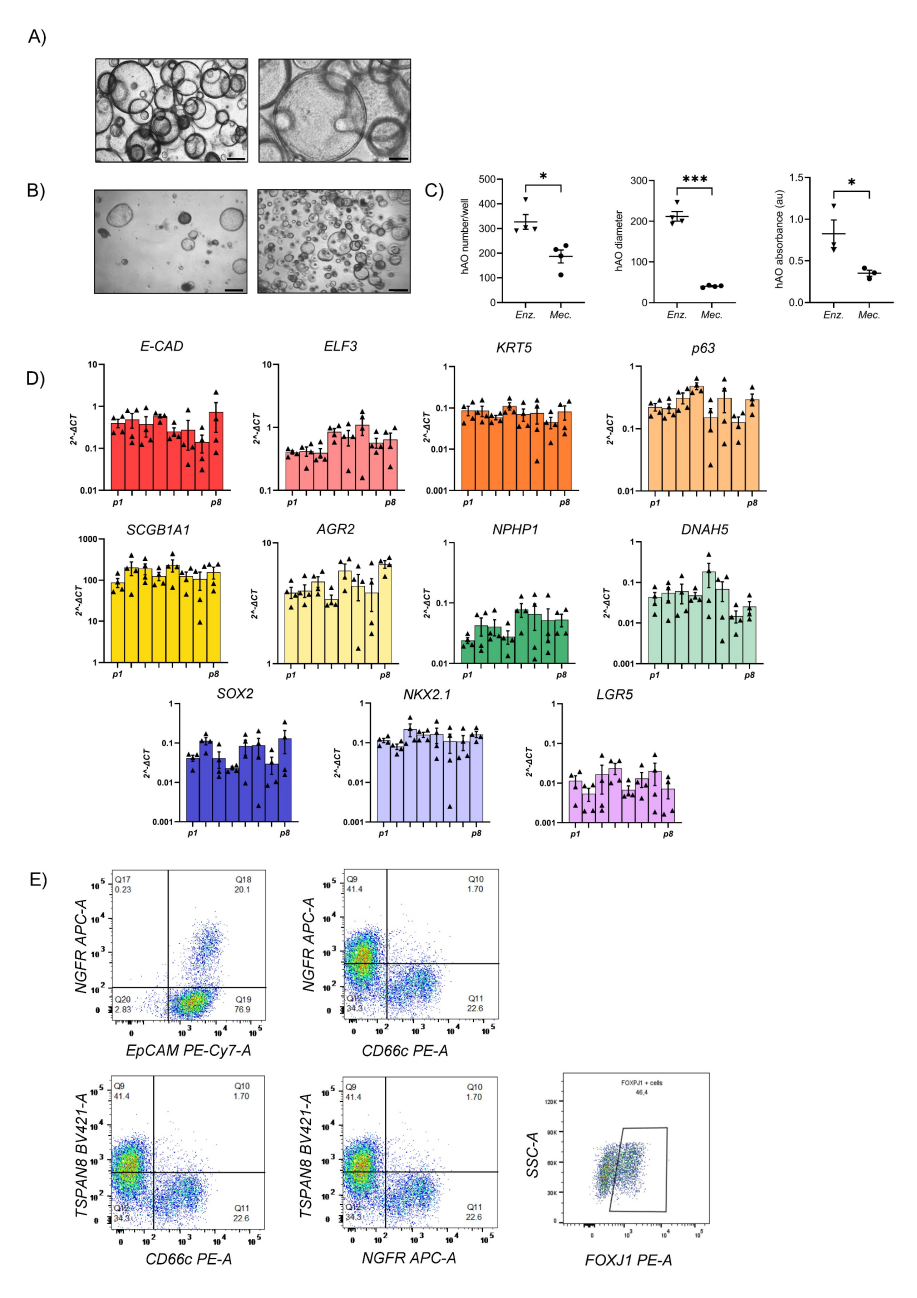
** hAO characterization.** (A) Representative bright-field images of hAO after 21 days from seeding. Scale bars 200µm (left) and 90µm (right). Images were acquired using a DS-Fi3 Nikon camera; (B) Representative bright-field images of hAO in two different seeding protocols: from mechanically (left) to enzymatic dissociation (right). Images were acquired using a DS-Fi1 Nikon camera. Scale bars 200µm for both images; (C) Quantification of organoid number (left), diameter in µm (middle) and viability (right) comparing the two different seeding protocols. The data reported in the graphs are mean± SEM (n=4; n=3 for viability). Statistical analysis was performed using two-tailed unpaired t test with Welch's correction for organoid number and diameter analysis (*p<0.05, ***p<0.001); two-tailed unpaired t test for viability analysis (*p<0.05); (D) Gene expression levels of different epithelial markers demonstrated the airway feature of organoids during passages, from p1 to p8. The data reported in the graphs (log_10_) are mean *±* SEM of 2^-*∆*CT of different organoid cell lines (n=4); (E) Representative flow cytometry dot plots of hAO surface and intracellular airway markers at 21 days after seeding (n=6). Antibodies reported in Table 2.

hAO were generated from biopsy specimens obtained from 15 healthy lung donors (Fig. Supplementary 1C). Organoids presented a spherical shape and an inner cavity with a diameter ranging from 30µm to 450µm (Fig. 1A). We improved the hAO culture technique by replacing the mechanical dissociation step with an enzymatic protocol that enabled faster growth and larger organoids than described previously (Fig. 1B) 11. The reliability of enzymatic dissociation was supported by quantitative analysis showing number of organoids, average size, and cell viability, as assessed by MTT assay (Fig. 1C).

### Airway signature

**Figure 2:**
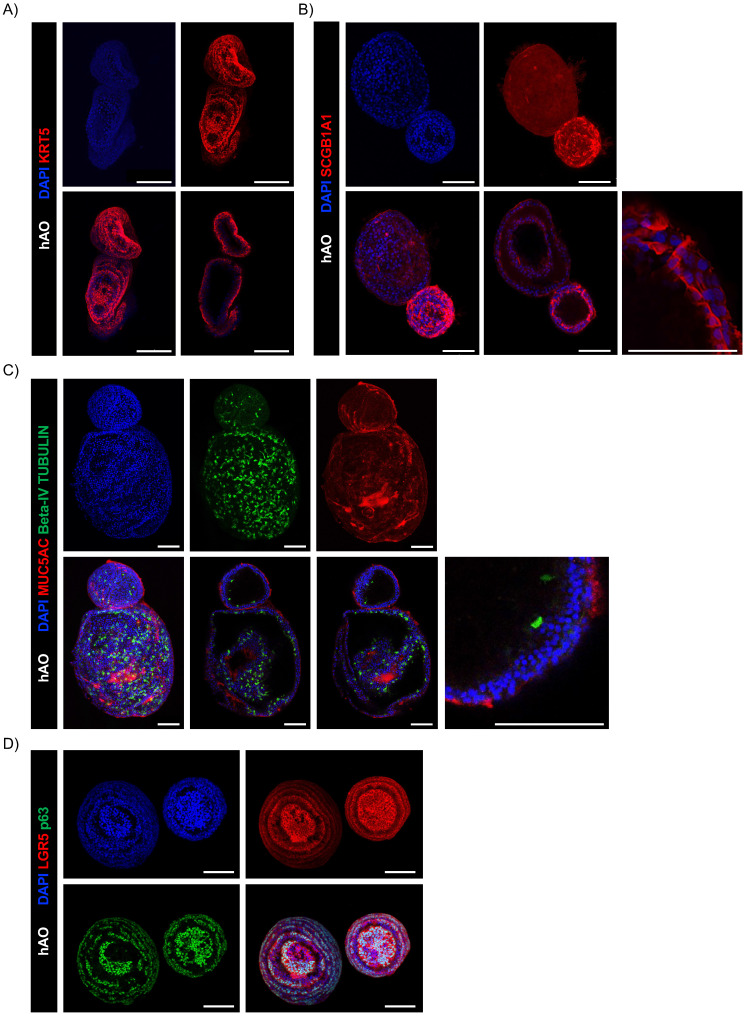
** Confocal microscopy images of hAO.** Representative confocal microscopy z-stacks and maximum projections of hAO (n=4), objective 20X (Leica SP8). Scale bar 100µm. (A) KRT5 (in Alexa Fluor-555); (B) SCGB1A1 (in Alexa Fluor-555); C) MUC5AC (in Alexa Fluor-555) and beta-IV Tubulin (in Alexa Fluor-488); (D) LGR5 (in Alexa Fluor-555) and p63 (in Alexa Fluor-488).

The gene expression profiles of hAO derived from distinct donors were similar during respective passages and recapitulated those of distinct airway tissue cell types, thus proving the reproducibility of this culture method. We demonstrated that mRNAs encoding E-cadherin (E-Cad) and E74-like factor 3 (ELF3), biomarkers of generic airway epithelial cells 23-25, were present during passages. Basal cells were identified by expressions of keratin-5 (KRT5) and tumor protein 63 (p63), which were constant during different passages (Fig. 1D). Club cell marker secretoglobin family 1A member 1 (SCGB1A1), also known as club cell secretory protein 10 (CC10), was very highly expressed in comparison to other markers, indicating the maintenance of airway integrity and repair of organoids 26. Furthermore, anterior gradient protein 2 homolog (AGR2), one of the secretory goblet cell markers, was highly expressed. Ciliary markers nephrocystin 1 (NPHP1) and dynein axonemal heavy chain 5 (DNAH5) were present and both were expressed continuously during passages (Fig.1D). SRY-box 2 (SOX2) and NK2 homeobox 1 (NKX2.1), key markers of lung progenitor cells, were consistently expressed throughout the passages. Interestingly, leucine-rich repeat-containing G-protein coupled receptor 5 (LGR5) was also stably maintained (Fig. 1D). Flow cytometry confirmed airway identity by demonstrating typical surface and intracellular markers such as EpCAM (92,08 ± 2,33), CD271 (32,86 ± 5,92), CD66c (55,99 ± 12,73), tetraspanin-8 (TSPAN8) (21,07 ±12,02), and forkhead box protein J1 (FOXJ1) (36,35 ± 5,42) that characterize basal, secretory, and ciliated cells (Fig. 1E and Supplementary 1D). The same signature was observed through confocal microscopy, which additionally demonstrated the specific spatial distribution of these cells that closely mimicked* in vivo* epithelial organization (Fig. 2A-C). To further confirm the airway identity of our hAO, an immunohistochemical assay using a monoclonal antibody highly specific to the human lung alveolar type 2 cell constantly yielded negative results (data not shown) 27,28. Notably, LGR5, an important marker of adult stem cells and critical modulator of their activity 29, co-localized with p63 (Fig. 2D).

**Figure 3:**
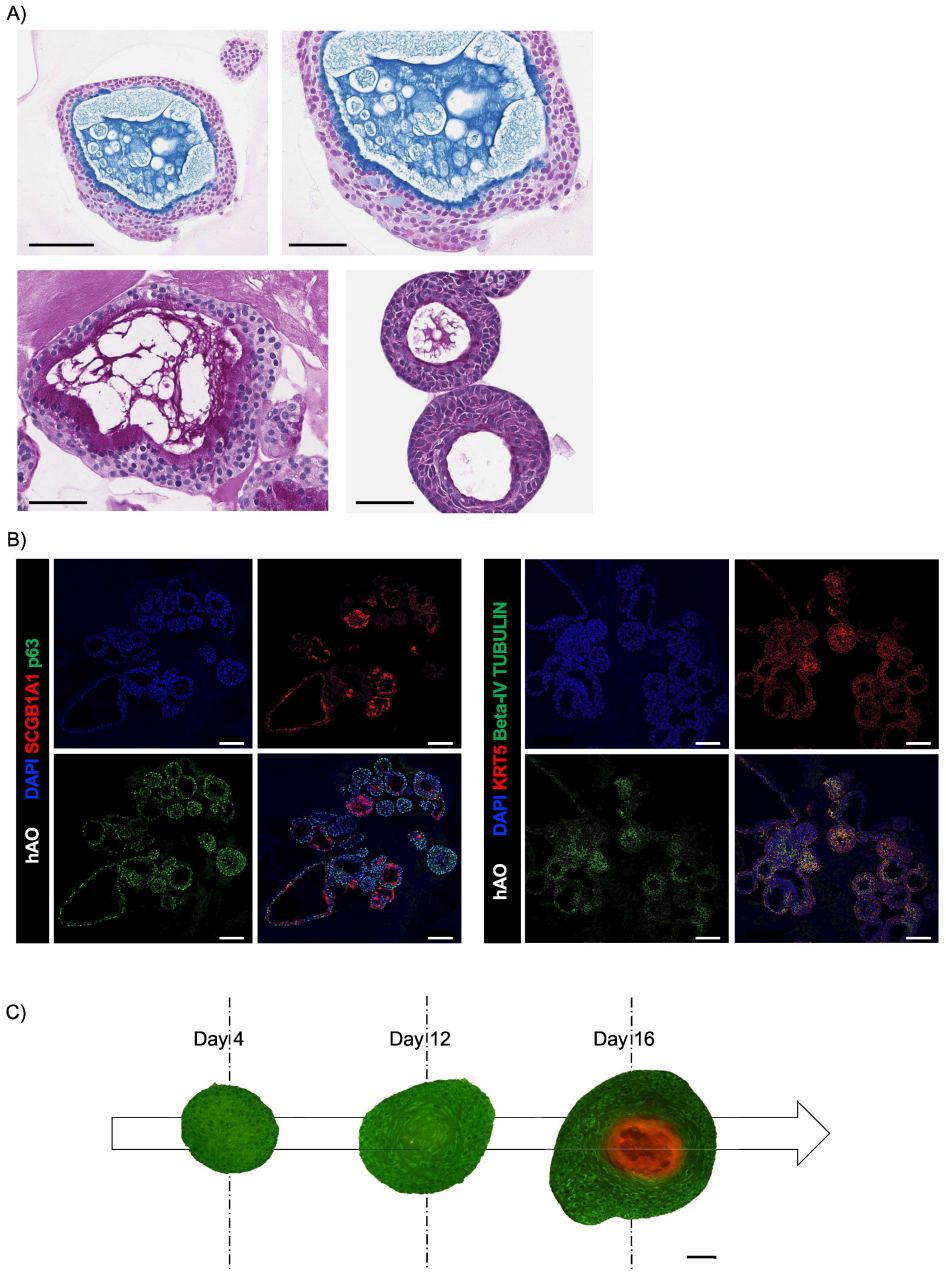
** Histological and immunohistochemical analysis of hAO. (A)** Representative images of AB (upper panel)-PAS (lower panel) sections of hAO (3μm thickness, n=3). Objective 20X and 40X (Leica Aperio AT2). Scale bars 200µm and 60µm; (B) Representative confocal microscopy images of hAO sections, objective 20X (Leica SP8). Scale bar 100µm. Left panel: SCGB1A1 (in Alexa Fluor-555) and p63 (in Alexa-488). Right panel: KRT5 (in Alexa Fluor-555) and beta-IV Tubulin (in Alexa Fluor-488). (D) Representative MTP stack label-free images (500x500x100µm) of hAO at day 4, day 12 and day 16 after seeding. Scale bar 100µm (Leica Stellaris DIVE 8).

Next, we sought to determine whether our hAO were organotypic and thus able to mimic the cellular composition, histoarchitecture, and functionality of the tissue of origin. AB-PAS staining sections of hAO at different passages (p4 to p8) showed acidic and neutral mucins released by secretory cells into the organoid lumen (Fig. 3A). To ensure a spatially-matched evaluation, reduce inter-sample variability, and enhance the reliability of our findings, we used the same histological sections to allow a direct correlation between mucus production and specific cellular markers. As shown in Fig. 3B, basal cells (p63+ and KRT5+) were in the outer epithelial layer of the organoids, while secretory cells (SCGB1A1+) were in the luminal surface and ciliated cells (beta-IV Tubulin+) were in both apical and luminal surfaces.

### MTP microscopy of hAO

MTP microscopy enabled the examination of cellular structures and properties while preserving the organoids' natural environments. To analyze the morphological changes and the modification of cell shapes during growth, we performed an MTP living-analysis. Four days after seeding, hAO displayed a reticular organization, with nuclei encircled by filamentous structures. hAO size ranged between 10-20 μm. On day 12 after seeding, clusters of nuclei formed a circular shape, and hAO sizes ranged from 50 to 100μm. On day 16, two autofluorescence signals were detected, indicating the presence of specific cell populations. At this stage, the organoids underwent significant morphological changes, growing to sizes of approximately 200-300μm. Nuclei formed a circular pattern, with a noticeable thickening of the cells located on the organoid exterior. Additionally, the presence of mucin in the central lumen suggested functional differentiation (Fig. 3C, shown in orange).

### Generation and characterization of human lung fibroblasts

**Figure 4:**
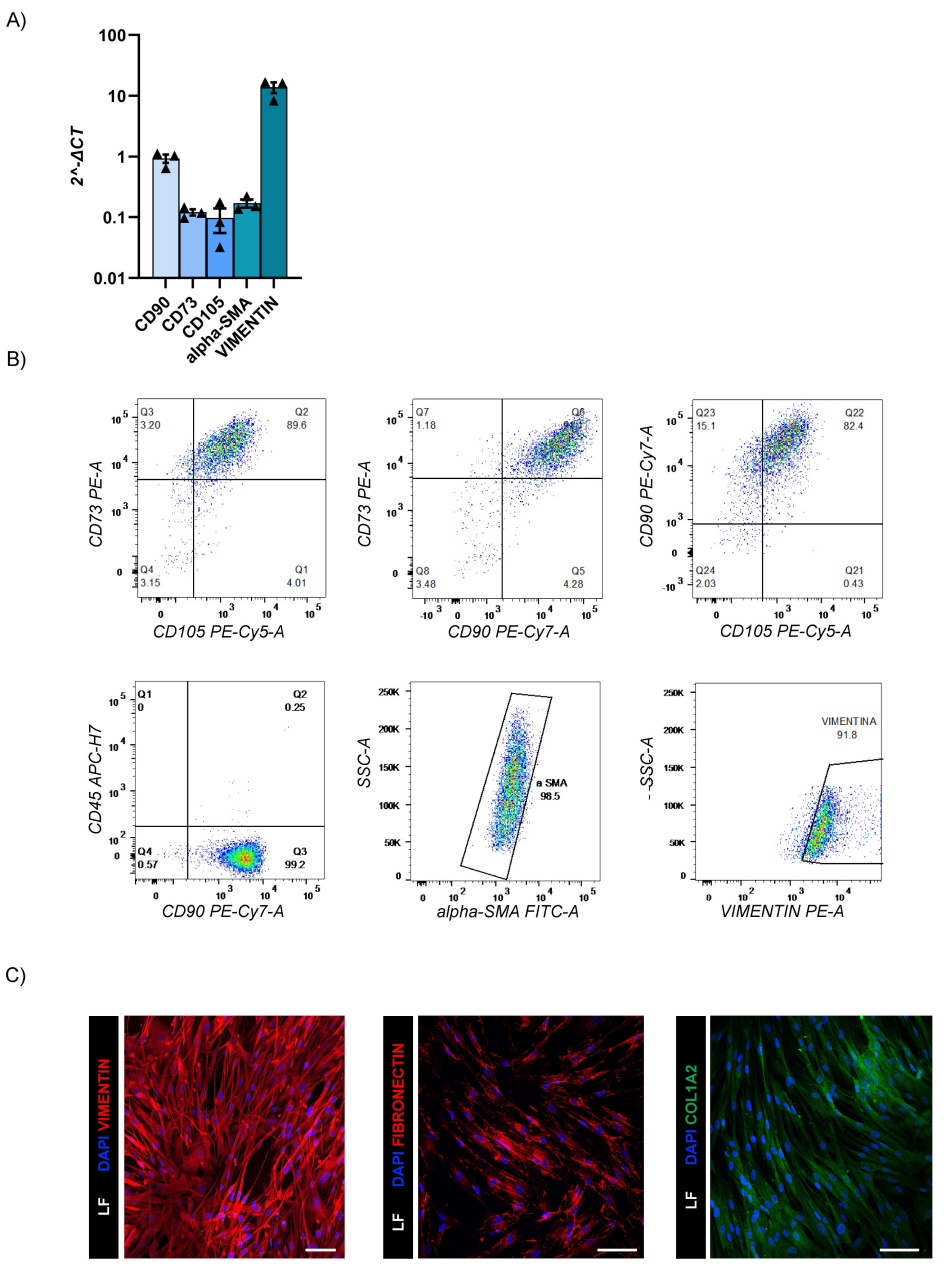
** LF characterization.** (A) Gene expression levels of fibroblast markers. The data reported in the graphs (log10) are mean ± SEM of 2^-∆CT of different LF donors (n=3); (B) Representative flow cytometry dot plots of LF surface and intracellular markers (n=3); (C) Representative confocal microscopy images of intracellular vimentin (Alexa Fluor-555), fibronectin (Alexa Fluor-555) and COL1A2 (Alexa Fluor-488) markers (n=3). Scale bar 100µm (Leica SP8).

LF were successfully isolated from the same healthy lung donor biopsy specimens. These primary cell lines were fully characterized by RT-qPCR and flow cytometry that revealed expressions of CD90, CD73, CD105, alpha-SMA, and vimentin; and negativity for CD45 (Fig. 4A-C and Suppl. 2B). The detection of ECM-specific markers such as COL1A2 and fibronectin by flow cytometry and immunofluorescence staining confirmed the ability of our LF to support the secretion of ECM (Fig. 4B-C).

### Generation and characterization of human airway assembloids

To create a model that more closely resembles the natural organ, we developed an assembloid that replicates healthy airway structure. First, we investigated the ability of synthetic ECMs to support the formation of a 3D structure, but failed to establish genuine intercellular communication. Therefore, we used innovative devices to build and support rapid cell aggregation with high reproducibility, and omitted plate centrifugation to reduce cellular stress. We therefore selected a specific low-attachment U-bottom plate coated with a fully xenofree polymer that avidly facilitates cellular interactions. This approach avoided the use of soluble ECM and yielded a scaffold-free system (Fig. Supplementary 2C).

**Figure 5:**
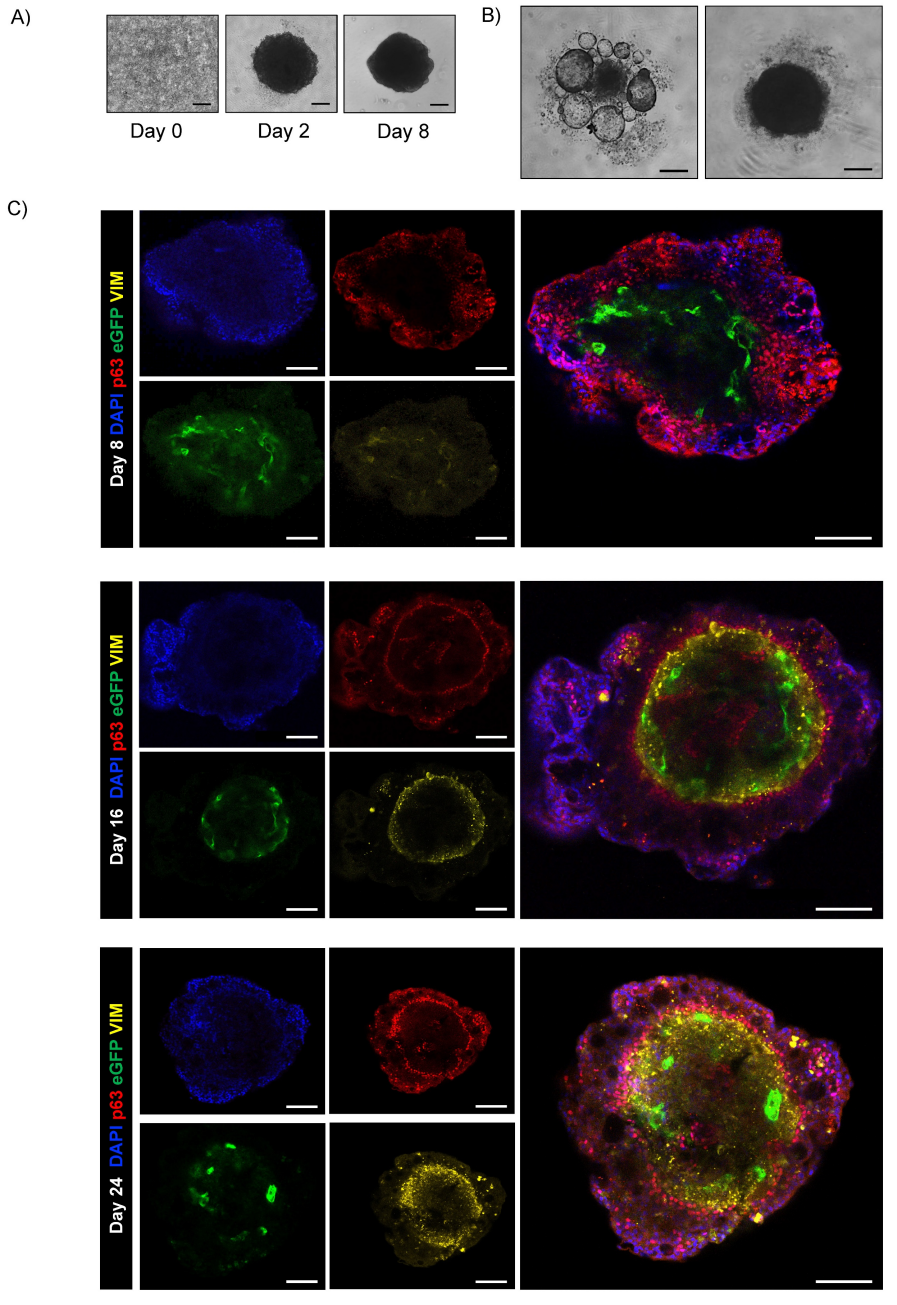
** Airway assembloid generation and characterization.** (A) Representative bright-field images of human airway assembloids at different days from seeding. Scale bars 200µm. Images were acquired using a DS-Fi3 Nikon camera; (B) Representative bright-field images of hAO with HUVEC/TERT2 (left) and hAO with LF (right). Scale bars 200µm. Images were acquired using a DS-Fi1 Nikon camera; (C) Representative confocal microscopy images of human airway assembloids at different time points: day 8, day 16 and day 24 (n=6). Basal cells represented by p63 (in red), LF by vimentin (in yellow) and HUVEC/TERT2 eGFP+ (in green). Objective 20X (Leica SP8). Scale bar 100µm.

The monitoring of assembloids from initial seeding enabled assessment of the interplay between epithelial and stromal compartments and investigation of the chronology and dynamics of cluster formation (Fig. 5A). First, we observed that the fibroblast compartment was essential for driving the formation of an organized 3D structure by supporting ECM secretion 30. In contrast, when only epithelial and endothelial components were present, no structured assembly occurred (Fig. 5B). Confocal microscopy on days 8, 16, and 24 disclosed the resemblance of the assembloid 3D structure to that of natural airways, the interactive organization of differentiated cell types and epithelial function.

**Figure 6:**
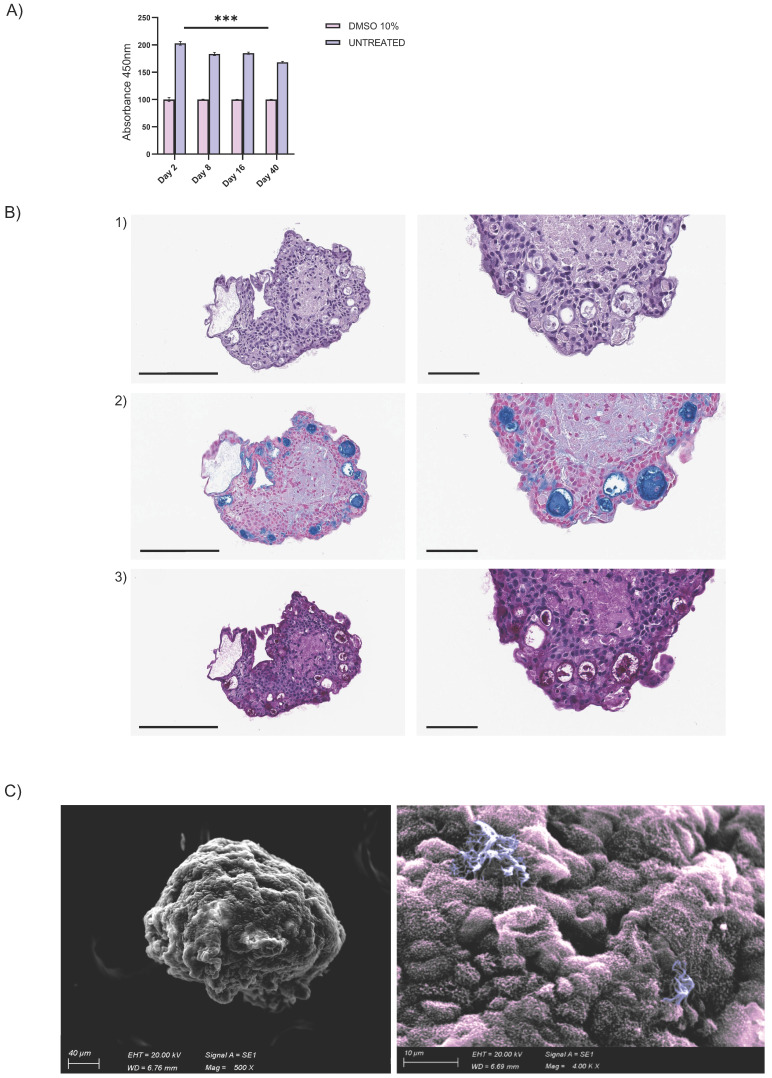
** Airway assembloid viability and functionality.** (A) Assembloid viability test at different time points up to 40 days. Assembloids were treated with DMSO 10% for 48h as control. The data reported in the graphs are mean *±* SEM of different assembloids (n=3); statistical analysis was performed using two-tailed unpaired t test (***p<0.001); (B) Representative images of H&E (panel 1) and AB (panel 2)-PAS (panel 3) sections of human airway assembloids at day 16 (3μm thickness). Objective 20X and 40X (Leica Aperio AT2). Scale bars respectively 200µm and 60µm; (C) Representative SEM image (left panel) and pseudo-coloured image (detail, right panel) of human airway assembloids at day 24, showing irregularly shaped multiciliated (violet) and nonciliated cells with microvilli (pink) (SEM EVO 10, Zeiss).

Endothelial cells were organized in the luminal surface and were surrounded by stromal cells, while epithelial cells formed an outer layer, resembling a basement membrane (Fig. 5C and Fig. Supplementary 2D). Furthermore, our assembloids remained viable for up to 40 days, maintaining good cell viability, structural integrity, and a well-preserved microenvironment (Fig. 6A).

Summarizing the key functions of the tissue of origin is as relevant as viability to prove that this avatar is not an inactive reproduction but a true mini-organ. The demonstration of mucus production and ciliary movement confirmed assembloid functionality and activity, thus validating that our model is biologically relevant and well-maintained in culture. This was also supported by H&E and AB-PAS staining and by SEM, which, as previously performed for the hAO, exhibited both basal and secretory cells (Fig. 6B-C). Additionally, ciliated cells within cystic airway organoids (Video 1S) and assembloids (Video 2S and Video 3S) disclosed both apical and luminal orientations, facilitating their motility within the ECM and further emphasizing the dynamic nature of the model 31.

**Figure 7:**
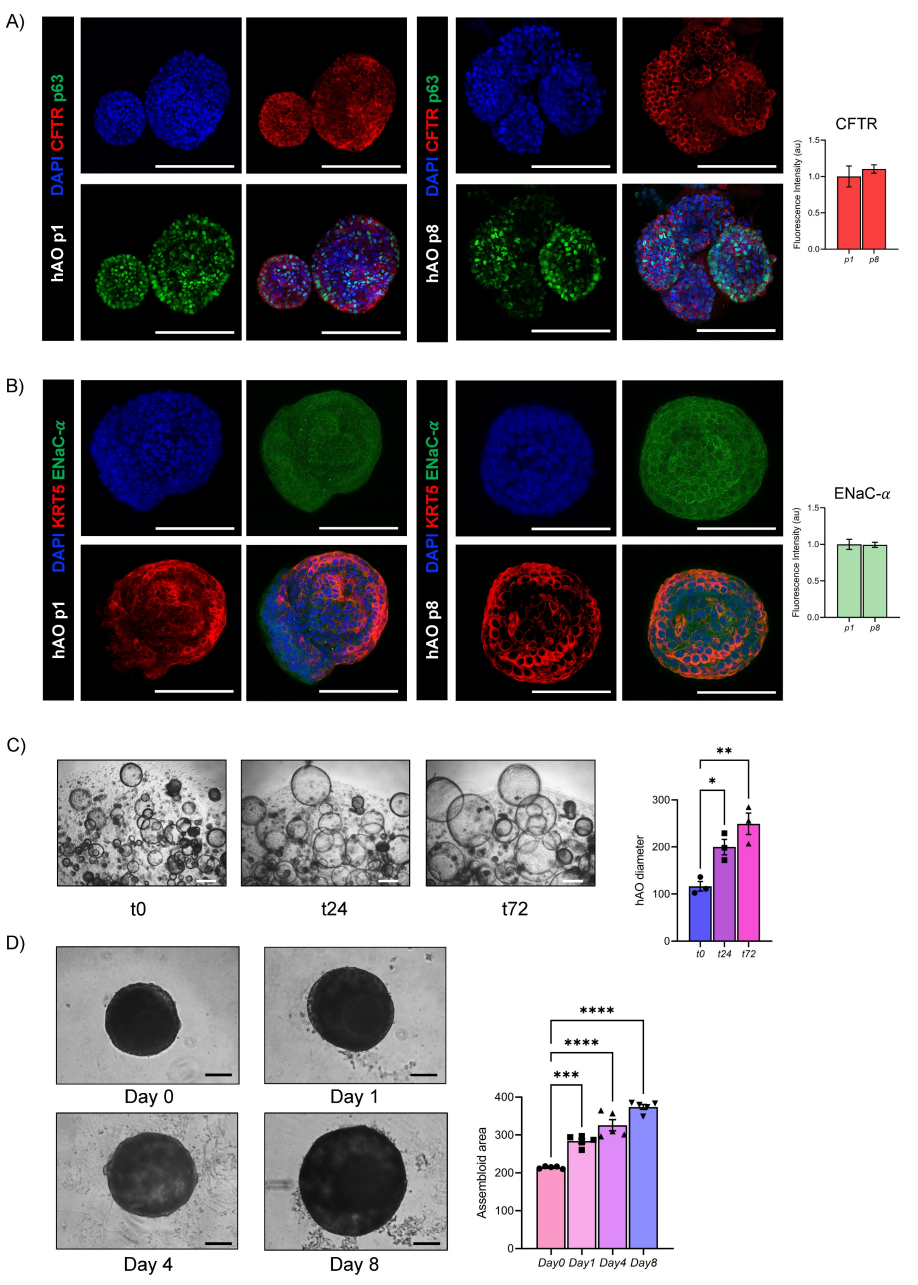
** Functional evaluation of airway epithelial physiology.** (A) Representative confocal microscopy maximum projections of hAO, objective 20X (Leica SP8). Scale bar 100µm. CFTR (in Alexa Fluor-555) and p63 (in Alexa Fluor-488) (left); quantification of mean fluorescence intensity with background subtraction (right). The data are presented as mean ± SEM (n=3 at passages 1 and 8). (B) Representative confocal microscopy maximum projections of hAO, objective 20X (Leica SP8). Scale bar 100µm. KRT5 (in Alexa Fluor-555) and ENaC-α (in Alexa Fluor-488) (left); quantification of mean fluorescence intensity with background subtraction (right). The data are presented as mean ± SEM (n=3 at passages 1 and 8). (C) Representative bright-field images of hAO at t0, t24, t72 after forskolin treatment (20µM) were acquired using a DS-Fi1 Nikon camera. Scale bars 200µm (left). Quantification of organoid diameter (in µm) at different timepoints after treatment (right). The data reported in the graphs are mean ± SEM (n=3). Statistical analysis was performed using one-way ANOVA, Dunnett's multiple comparison test vs t0 (*p<0.05 t0 vs t24, **p<0.01 t0 vs t72) (D) Representative bright-field images of airway assembloids at day 0, day 1, day 4, day 8 after forskolin treatment (20µM) were acquired using a DS-Fi1 Nikon camera. Scale bars 200µm (left). Quantification of the assembloid area at different timepoints after treatment (right). The data reported in the graphs are mean ± SEM (n=3). Statistical analysis was performed using one-way ANOVA, Dunnett's multiple comparison test vs Day 0(*p<0.05 of t0 vs t24, **p<0.01 t0 vs t72).

To further demonstrate the functionality of the airway epithelial component, we analyzed key epithelial ion channels at both transcriptional and protein levels in hAO. For the ENaC, the alpha-subunit, indispensable in the maintenance of fluid homeostasis in the lung, was tested 32. Particularly, CFTR and ENaC-α were stable during passages, from p1 to p8, as demonstrated by gene expression and confocal imaging analysis (Fig.7A, 7B, Fig. Supplementary 3C), Moreover, to assess the functional activity of CFTR we performed the forskolin-induced swelling assay, which demonstrated a significant increase in organoid size over time, confirming active ion and fluid transport (Fig. 7C).To validate the functional integrity of the airway epithelium in the assembloid, the same forskolin administration was performed and a progressive and significant increase in size over time was observed (Figure 7D).

## Discussion

New and advanced models will not only elucidate the pathobiology of pulmonary disorders but may also accelerate therapy development. Lung organoids represent easier-to-use and more ethically sustainable alternatives to animal models while closely simulating human pulmonary physiology and preserving the genetic and phenotypic features of their native tissue 33. Although animal models remain the primary tools for studying the pathobiology of lung diseases such as pulmonary fibrosis, chronic obstructive pulmonary disease, lung cancer, and play a key role in preclinical drug screening, their high cost, lengthy experimental timelines, and variability often lead to poor reproducibility and limited translational potential 34. Moreover, aligning with the European 3Rs principle (Replacement, Reduction, and Refinement), these models may minimize animal use in research 35-37.

In 2009, Rock et al. used murine adult airway stem cells to establish the first airway organoid culture protocol 38. A decade later, in 2019, Sachs and colleagues developed a long-term airway organoid culture system 21. Over the last decade, airway organoid technology has emerged as a versatile research tool for replicating the diversity of human airway pseudostratified epithelia and facilitating the *in vitro* study of neoplastic and infectious pulmonary diseases 39.

In this study, we first optimized the generation and culture of airway organoids derived from donor biopsy specimens, thus advancing our previous work 11. This methodology, based on enzymatic dissociation, enables the precise counting of single cells that constitute organoids, ensuring reproducibility in the culture system. Our hAO consistently expressed typical markers of basal, secretory (including club and goblet), and ciliated cells during passages, as demonstrated by multiple techniques. Moreover, levels of SOX2 and NKX2.1 indicated the self-renewal capacity of these adult stem cells, promoting their differentiation and supporting the identity and function of epithelial cells.

We detected LGR5 expression, a biomarker of stemness, during hAO passages. LGR5 is essential for self-renewal and differentiation, it is a key marker of active stem cells in organoids and unequivocally identifies self-renewing organoids 13. Indeed, its expression prolongs the stability of the model and maintains tissue regenerative capacity.

The most crucial indicator of functionality is mucus secretion 40, which was demonstrated not only by standard methods but also through the application of the most advanced and innovative technologies. Indeed, mucus secretion was further confirmed by MTP microscopy and label-free images that are advantageous because of their preservation of natural cellular structure and function while reducing interference and artifacts from dyes. This ground-breaking approach revealed ultrastructural details that included circular patterns of nuclear organization and sequential mucus secretion, suggesting the functional commitment of the organoids.

Although organoids are the most widely used 3D models in tissue modeling, they still exhibit limitations, particularly in establishing complex interactions between tissue cell types. Limited information regarding vascular structures, their interactions with the microenvironment, and inter-organ signaling continue to compromise their fidelity to human organs 41. A transition from the use of organoids to more complex networking structures such as assembloids is needed to facilitate studies of the interactions between multiple cell types and to thereby better understand pathobiology.

Assembloids are 3D cell culture systems including complex and integrative models that incorporate multiple types of organoids or specialized cell types. These structures are self-organizing, and thereby create a more comprehensive and dynamic system that better simulates interactions within or between organs. Assembloids are useful for investigating tissue remodeling, complex diseases, and multi-tissue dynamics 42. Consequently, assembloids are increasingly recognized as a better alternative in multiple fields of biomedical research, and lead the advancement of stem-cell research. Recent studies have focused on assembloid generation through multicellular co-culture and 3D extrusion printing. This technology has been applied to study normal tissues of the brain and gastrointestinal tract 43-46 as well as bladder and small cell lung cancers 47,48.

Based on these advancements, we developed a new 3D model of healthy lung tissue using an experimental methodology that enabled the self-aggregation of hAO in a more complex structure through interactions between primary lung fibroblasts and an endothelial cell line. Organoids typically grow in solid basement membrane extracts, with Matrigel being one of the most used options due to its ECM proteins that include laminin, collagen type IV, proteoglycans, and growth factors. To create a vital, easy-to-use, and simply reproducible model, we generated our avatar without the use of a synthetic ECM. Lung fibroblasts included in these structures secreted ECM proteins such as COL1A2 and fibronectin. Interestingly, we observed that the stromal component drove appropriate assembloid formation, while the presence of only epithelial and endothelial cells did not generate an organized model. These experiments confirmed the crucial role of stromal cells in the formation of organized models such as assembloids 49,50. Furthermore, our approach obviated the use of scaffolds, which are disadvantageous in modeling because of their variability, non-human origin, and potential lack of biological relevance in human tissue systems.

The functions of endothelial cells go beyond their role as structural components of vasculature 51. Our goal was to incorporate endothelial cells into our 3D system to recreate the *in vivo* environment. However, the isolation of endothelial cells from human lung tissue is difficult due to the coexistence of multiple cell types. After several attempts that yielded a high degree of contamination by non-endothelial cells such as fibroblasts as demonstrated previously by Gaskill et al. 52, we decided to use HUVEC, widely recognized for their key role in angiogenesis 53, to promote the vascular signature within the assembloid model. In addition, the used hTERT-transfected HUVEC cell line has been demonstrated to maintain endothelial characteristics at both mRNA and protein levels similar to those of standard primary HUVEC 54.

The functionality of our assembloid is crucial for its validation as a useful airway model. After assembly that incorporated fibroblasts and endothelial cells, our 3D avatar demonstrated expressions of typical airway markers and key functions of the airway epithelium such as ciliary beating observed by microscopy and mucus production demonstrated by AB-PAS staining.

Our selection of the transcription factor p63 to label the epithelial component deserves further comment. This marker was chosen because it identifies basal cells, which are relatively undifferentiated cells of the pulmonary pseudostratified mucociliary epithelium 38,55. The use of p63 ensured the identification of basal cells and their persistence through multiple passages. This was crucial to our study because basal cells constitute the most important cell type, without which the lung cannot generate other essential cells. In addition, p63-positive cells serve not only as airway stem cells, but also enable assembloid functionality by producing ciliated and mucosecretory cells. The final and certainly crucial parameter of our 3D assembloid is its long-term viability.

To complement the structural characterization of this 3D model and ensure its physiological relevance, we performed a molecular and functional evaluation of ion transport mechanisms known to be central to airway epithelial integrity and homeostasis.

Particularly, we demonstrated that from early to late passages airway organoids maintain stable gene and protein expression levels and functional activity of CFTR and ENaC-α, thus confirming that ion homeostasis is physiologically preserved in this system 56. Interestingly, the functional relationship between the ion channels was not altered even when the airway organoids were integrated in the 3D assembloid structure.

In conclusion, we propose a reproducible 3D healthy airway assembloid as an advanced *in vitro* platform for the study of morphology, functions, and cellular interactions within the lung.

### Limitations

Although our 3D lung assembloid model represents a significant advance in lung research, some aspects require optimization. The inclusion of immune cells could further enhance its applicability in studying pulmonary inflammation, while more sophisticated vascularization strategies may improve its recapitulation of native lung microcirculation. Additionally, the absence of airflow dynamics and mechanical stimuli, key factors in lung physiology, could be addressed by integrating bioreactor systems. Despite these limitations, our model sustains viability, functionality, and key cellular interactions over time, thus offering a promising tool for studying lung diseases and testing new therapeutic approaches.

## Supplementary Material

Supplementary figures.

Video S1.

Video S2.

Video S3.

## Figures and Tables

**Table 1:** Primers

E-Cad (E-Cadherin)	FWD	TGCTCTTCCAGGAACCTCTG
	REV	GCGGCATTGTAGGTGTTCA
ELF3 (E74 like ETS transcription factor 3)	FWD	CTCTTCCCCAGCGATGGTTT
	REV	CCCTCGAGACAGTCCCAGTA
KRT5 (Keratin 5)	FWD	GCATCACCGTTCCTGGGTAA
	REV	GACACACTTGACTGGCGAGA
p63 (Transformation-related protein 63)	FWD	CCAGAGATGGGCAAGTCCTG
	REV	ACTGTCCGAAACTTGCTGCT
SCGB1A1 (Secretoglobin Family 1A member 1)	FWD	TCCTCCACCATGAAACTCGC
	REV	AGGAGGGTTTCGATGACACG
AGR2 (Anterior gradient protein 2 homolog)	FWD	TCAGAAGCTTGGACCGCATC
	REV	AGTGTAGGAGAGGGCCACAA
NPHP1 (Nephrocystin-1)	FWD	CAGAGCCACATGGCAACCTA
	REV	ACCCAGCCACAGCTTAACTC
DNAH5 (Dynein axonemal heavy chain 5)	FWD	AGAGGCCATTCGCAAACG
	REV	CCCGGAAAATGGGCAAACTG
SOX2 (SRY-sex determining region Y-box 2)	FWD	CACATGAAGGAGCACCCGGATTAT
	REV	CGGGAAGCGTGTACTTATCCTTCT
NKX2.1 (NK2 homebox 1)	FWD	ACCAAGCGCATCCAATCTCA
	REV	CAGAGCCATGTCAGCACAGA
LGR5 (Leucine-rich repeat-containing G protein coupled receptor 5)	FWD	AGGCCTTGTCTGGGTTGAA
	REV	TGGCTTCACTGGGTACTGTTT
CD73 (Cluster of differentiation 73)	FWD	CACTGGGACATTCGGGTTTT
	REV	CGTCCACACCCCTCACTTTC
CD90 (Cluster of differentiation 90)	FWD	CGAACCAACTTCACCAGCAAAT
	REV	CCTTGCTAGTGAAGGCGGATA
CD105 (Cluster of differentiation 105)	FWD	CCGCGCTTCAGCTTCCT
	REV	GAGGGTGCCGGTTTTGG
VIM (Vimentin)	FWD	AGGCAAAGCAGGAGTCCACTGA
	REV	ATCTGGCGTTCCAGGGACTCAT
ACTA2 (α Smooth Muscle Actin 2)	FWD	CTGTTCCAGCCATCCTTCAT
	REV	TCATGATGCTGTTGTAGGTGGT
CFTR (Cystic Fibrosis Transmembrane Conductance Regulator)	FWD	CACAGGACAGCCCTTCTTTC
	REV	TGCCCATGGCCTATCTACTT
ENaC - α (Epithelial Sodium Channel - α)	FWD	GCTGATAACCAGGACAAAACACAA
	REV	CGTCGCTGGGCAGGAA

**Table 2:** Antibodies

Antibody Target	Host Animal/species	Fluorophore(s)/Conjugate	Clone	Cat. Number	Vendor/ company
anti-EpCAM	Mouse	PECy7	1B7	25-9326-42	eBioscience
anti-CD271	Mouse	Alexa 647	C40-1457	560326	BD
anti-CD66c	Mouse	PE	KOR-SA3544	12-0667-42	eBioscience
anti-TSPAN8	Rat	Alexa 405	458811	FAB4734V	R&D
anti-FOXJ1	Mouse	-	2A5	14-9965-82	eBioscience
anti-CD90	Mouse	PECy7	5E10	561558	BD
anti-CD73	Mouse	PE	AD2	550257	BD
anti-CD105	Mouse	PerCP-Cy5.5	266	560819	BD
anti-Vimentin	Mouse	-	VIM 3B4	CBL202-K	Merck-Chemicon
anti-p63	Rabbit	-	EPR5701	ab124762	Abcam
anti-CC10/SCGB1A1	Mouse	-	E-11	sc-365992	Santa Cruz
anti-KRT5	Mouse	-	XM26	MA5-12596	Invitrogen
anti-beta IV tubulin	Rabbit	-	EPR16775	ab179504	Abcam
anti-HT2-280	Mouse	-	N/A	TB27AHT2-280	Terrace Biotech
Anti-MUC5AC	Mouse	-	45M1	sc-21701	Santa Cruz
anti-CFTR	Mouse	-	CF3	NB300-511	Novus Biologicals
Anti-EnaC α	Rabbit	-	Polyclonal	orb101489	Biorbyt
					
Anti-rabbit	Goat	Alexa 555	-	AB-143157	Invitrogen
Anti-mouse	Goat	Alexa 488	-	Ab-2534069	Invitrogen
Anti-mouse	Goat	Alexa 555	-	A21-422	Invitrogen
IgG Isotype control	Mouse	-	-	31903	Invitrogen

## Abbreviations

3D: three-dimensional
2D: two-dimensional
hAO: human airway organoids
RT: room temperature
FBS: fetal bovine serum
LF: lung fibroblasts
SEM: scanning electron microscopy
H&E: hematoxylin and eosin
AB-PAS: Alcian-blue periodic acid Schiff
ECM: extracellular matrix
MTP: Multi-photon
HUVEC: human umbilical vein endothelial cells
LV: lentivirus
GFP: green fluorescence protein
TU: transducing unit
HMDS: hexamethyldisilazane
SEM: standard error of the mean
SCGB1A1: secretoglobin family 1A member 1
CC10: club cell secretory protein
AGR2: anterior gradient protein 2 homolog
NPHP1: ciliary markers nephrocystin 1
DNAH5: dynein axonemal heavy chain 5
SOX2: SRY-box 2
NKX2.1: NK2 homeobox 1
LGR5: leucine-rich repeat-containing G-protein coupled receptor 5
TSPAN8: tetraspanin-8
FOXJ1: forkhead box protein J1
KRT5: keratin-5
p63: tumor protein 63
E-Cad: E-cadherin
ELF3: E74-like factor 3
CFTR: cystic fibrosis transmembrane conductance regulator
α-ENaC: epithelial sodium channel alpha subunit

## References

[1] Nadkarni RR, Abed S, Draper JS (2016). Organoids as a model system for studying human lung development and disease. Biochem Biophys Res Commun.

[2] Bissell MJ, Radisky D (2001). Putting tumours in context. Nat Rev Cancer.

[3] Beumer J, Clevers H (2021). Cell fate specification and differentiation in the adult mammalian intestine. Nat Rev Mol Cell Biol.

[4] Bissell MJ, Kenny PA, Radisky DC (2005). Microenvironmental regulators of tissue structure and function also regulate tumor induction and progression: the role of extracellular matrix and its degrading enzymes. Cold Spring Harb Symp Quant Biol.

[5] Inman JL, Bissell MJ (2010). Apical polarity in three-dimensional culture systems: where to now?. J Biol.

[6] Hattori N (2014). Cerebral organoids model human brain development and microcephaly. Mov Disord Off J Mov Disord Soc.

[7] Takebe T, Zhang R-R, Koike H (2014). Generation of a vascularized and functional human liver from an iPSC-derived organ bud transplant. Nat Protoc.

[8] Dossena M, Piras R, Cherubini A (2020). Standardized GMP-compliant scalable production of human pancreas organoids. Stem Cell Res Ther.

[9] Loomans CJM, Williams Giuliani N, Balak J (2018). Expansion of Adult Human Pancreatic Tissue Yields Organoids Harboring Progenitor Cells with Endocrine Differentiation Potential. Stem Cell Rep.

[10] Dye BR, Hill DR, Ferguson MAH (2015). In vitro generation of human pluripotent stem cell derived lung organoids. eLife.

[11] Winkler AS, Cherubini A, Rusconi F (2022). Human airway organoids and microplastic fibers: A new exposure model for emerging contaminants. Environ Int.

[12] Yao Q, Cheng S, Pan Q (2024). Organoids: development and applications in disease models, drug discovery, precision medicine, and regenerative medicine. MedComm.

[13] Zhao Z, Chen X, Dowbaj AM (2022). Organoids. Nat Rev Methods Primer.

[14] Kühl L, Graichen P, von Daacke N (2023). Human Lung Organoids—A Novel Experimental and Precision Medicine Approach. Cells.

[15] Vazquez-Armendariz AI, Tata PR (2023). Recent advances in lung organoid development and applications in disease modeling. J Clin Invest [Internet].

[16] Thangam T, Parthasarathy K, Supraja K (2024). Lung Organoids: Systematic Review of Recent Advancements and its Future Perspectives. Tissue Eng Regen Med.

[17] Pașca SP (2018). The rise of three-dimensional human brain cultures. Nature.

[18] Miura Y, Li M-Y, Revah O, Yoon S-J, Narazaki G, Pașca SP (2022). Engineering brain assembloids to interrogate human neural circuits. Nat Protoc.

[19] Bouchard G, Zhang W, Ilerten I (2025). A quantitative spatial cell-cell colocalizations framework enabling comparisons between in vitro assembloids and pathological specimens. Nat Commun.

[20] Sikkema L, Ramírez-Suástegui C, Strobl DC (2023). An integrated cell atlas of the lung in health and disease. Nat Med.

[21] Sachs N, Papaspyropoulos A, Zomer-van Ommen DD (2019). Long-term expanding human airway organoids for disease modeling. EMBO J.

[22] Masters JR, Thomson JA, Daly-Burns B (2001). Short tandem repeat profiling provides an international reference standard for human cell lines. Proc Natl Acad Sci U S A.

[23] Post S, Heijink IH, Hesse L (2018). Characterization of a lung epithelium specific E-cadherin knock-out model: Implications for obstructive lung pathology. Sci Rep.

[24] Subbalakshmi AR, Sahoo S, Manjunatha P (2023). The ELF3 transcription factor is associated with an epithelial phenotype and represses epithelial-mesenchymal transition. J Biol Eng.

[25] Suzuki M, Saito-Adachi M, Arai Y (2021). E74-Like Factor 3 Is a Key Regulator of Epithelial Integrity and Immune Response Genes in Biliary Tract Cancer. Cancer Res.

[26] Hiemstra PS, Bourdin A (2014). Club cells, CC10 and self-control at the epithelial surface. Eur Respir J.

[27] Gonzalez RF, Allen L, Gonzales L, Ballard PL, Dobbs LG (2010). HTII-280, a Biomarker Specific to the Apical Plasma Membrane of Human Lung Alveolar Type II Cells. J Histochem Cytochem.

[28] Hoffmann K, Obermayer B, Hönzke K (2022). Human alveolar progenitors generate dual lineage bronchioalveolar organoids. Commun Biol.

[29] Leung C, Tan SH, Barker N (2018). Recent Advances in Lgr5+ Stem Cell Research. Trends Cell Biol.

[30] Kendall RT, Feghali-Bostwick CA (2014). Fibroblasts in fibrosis: novel roles and mediators. Front Pharmacol.

[31] Salgueiro L, Kummer S, Sonntag-Buck V (2022). Generation of Human Lung Organoid Cultures from Healthy and Tumor Tissue to Study Infectious Diseases. J Virol.

[32] Xu H, Chu S (2007). ENaC α-subunit variants are expressed in lung epithelial cells and are suppressed by oxidative stress. Am J Physiol-Lung Cell Mol Physiol.

[33] Clevers H (2016). Modeling Development and Disease with Organoids. Cell.

[34] Pampaloni F, Reynaud EG, Stelzer EHK (2007). The third dimension bridges the gap between cell culture and live tissue. Nat Rev Mol Cell Biol.

[35] Nizamoglu M, Joglekar MM, Almeida CR (2023). Innovative three-dimensional models for understanding mechanisms underlying lung diseases: powerful tools for translational research. Eur Respir Rev Off J Eur Respir Soc.

[36] Phan TH, Shi H, Denes CE (2023). Advanced pathophysiology mimicking lung models for accelerated drug discovery. Biomater Res.

[37] Neuhaus W Consensus Statement from the European Network of 3R Centres (EU3Rnet). ALTEX. 2020.

[38] Rock JR, Onaitis MW, Rawlins EL (2009). Basal cells as stem cells of the mouse trachea and human airway epithelium. Proc Natl Acad Sci U S A.

[39] Yang W, Li Y, Shi F, Liu H (2023). Human lung organoid: Models for respiratory biology and diseases. Dev Biol.

[40] Roe T, Talbot T, Terrington I (2025). Physiology and pathophysiology of mucus and mucolytic use in critically ill patients. Crit Care Lond Engl.

[41] Andrews MG, Kriegstein AR (2022). Challenges of Organoid Research. Annu Rev Neurosci.

[42] Kanton S, Paşca SP (2022). Human assembloids. Dev Camb Engl.

[43] Sun X, Kofman S, Ogbolu VC, Karch CM, Ibric L, Qiang L (2024). Vascularized Brain Assembloids With Enhanced Cellular Complexity Provide Insights Into the Cellular Deficits of Tauopathy. Stem Cells Dayt Ohio.

[44] Günther C, Winner B, Neurath MF, Stappenbeck TS (2022). Organoids in gastrointestinal diseases: from experimental models to clinical translation. Gut.

[45] Onesto MM, Kim J, Pasca SP (2024). Assembloid models of cell-cell interaction to study tissue and disease biology. Cell Stem Cell.

[46] Lin M, Hartl K, Heuberger J (2023). Establishment of gastrointestinal assembloids to study the interplay between epithelial crypts and their mesenchymal niche. Nat Commun.

[47] Kwak TJ, Lee E (2020). In vitro modeling of solid tumor interactions with perfused blood vessels. Sci Rep.

[48] Mei J, Liu X, Tian H (2024). Tumour organoids and assembloids: Patient-derived cancer avatars for immunotherapy. Clin Transl Med.

[49] Richards Z, McCray T, Marsili J (2019). Prostate Stroma Increases the Viability and Maintains the Branching Phenotype of Human Prostate Organoids. iScience.

[50] Kim E, Choi S, Kang B (2020). Creation of bladder assembloids mimicking tissue regeneration and cancer. Nature.

[51] Matsumoto K, Yoshitomi H, Rossant J, Zaret KS (2001). Liver organogenesis promoted by endothelial cells prior to vascular function. Science.

[52] Gaskill C, Majka SM (2017). A high-yield isolation and enrichment strategy for human lung microvascular endothelial cells. Pulm Circ.

[53] Porter AM, Klinge CM, Gobin AS (2011). Biomimetic hydrogels with VEGF induce angiogenic processes in both hUVEC and hMEC. Biomacromolecules.

[54] Mu X, Sang Y, Fang C (2015). Immunotherapy of tumors with human telomerase reverse transcriptase immortalized human umbilical vein endothelial cells. Int J Oncol.

[55] Bilodeau C, Shojaie S, Goltsis O (2021). TP63 basal cells are indispensable during endoderm differentiation into proximal airway cells on acellular lung scaffolds. Npj Regen Med.

[56] Konstas A-A, Koch J-P, Korbmacher C (2003). cAMP-dependent activation of CFTR inhibits the epithelial sodium channel (ENaC) without affecting its surface expression. Pflugers Arch.

