# Supplementary material for: Generation of Human 3D Airway Assembloids for Advanced Modeling
Source: Int J Biol Sci. 2025 Oct 1;21(14):6234–51. doi: 10.7150/ijbs.113920 (PMC12594573; doi:10.7150/ijbs.113920)

Figure Supplementary 1. (A) From lung biopsies to organoid 3D-culture: schematic representation of isolation process of hAO. Created in https://BioRender.com; (B) Schematic representation of isolation process of LF from lung biopsies created in https://BioRender.com. Representative bright-field images of LF acquired using a DS-Fi1 Nikon camera. Scale bars 200µm (left) and 90 µm (right); (C) Sex and gender of donors. (D) Negative controls of representative flow cytometry dot plots of hAO surface and intracellular airway markers at 21 days after seeding (n=6).

Figure Supplementary 2. (A) Schematic representation of lentivirus production, created in https://BioRender.com (upper panel). HUVEC/TERT2 were infected with eGFP expressing lentiviral vector as demonstrated by confocal microscopy (scale bars 200µm for bright-field image; 100µm for confocal microscopy image) (lower panel left) and flow cytometry analysis (lower panel right); (B) Negative controls of representative flow cytometry dot plots of LF surface and intracellular markers (n=3). (C) Schematic representation of seeding protocol of human airway assembloids created in https://BioRender.com; (D) Representative 3D image of human airway assembloid at day 16. Basal cells represented by p63 (in red), LF by vimentin (in yellow) and HUVEC/TERT2 eGFP+ (in green). Objective 20X (Leica SP8). Scale bar 100µm.

Figure Supplementary 3. (A) Representative STR profile of HUVEC/TERT2 confirming their identity as manufacturer's characterization (B). (C) Gene expression levels of CFTR and ENaC-α of hAO at passages 1, 4 and 8. The data reported in the graphs are mean ± SEM of $2^{-\Delta\Delta CT}$ of different hAO (n=4).

Figure Supplementary 1

A)

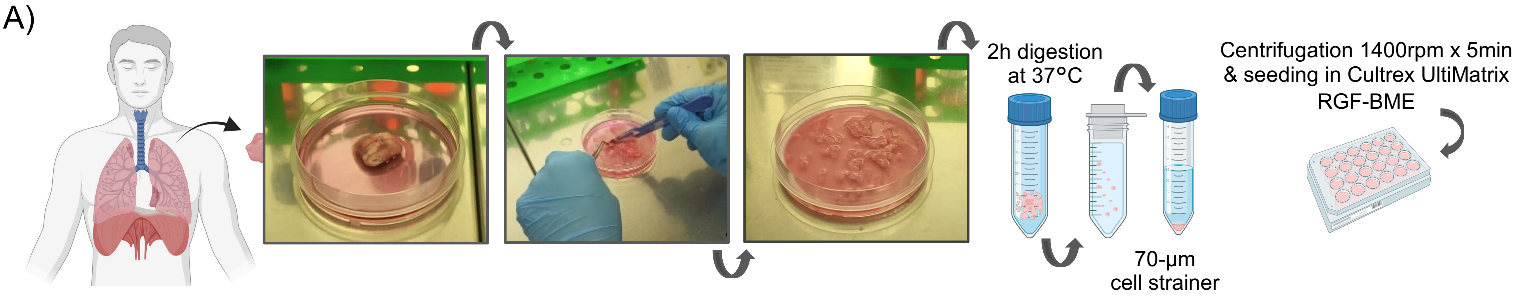

B)

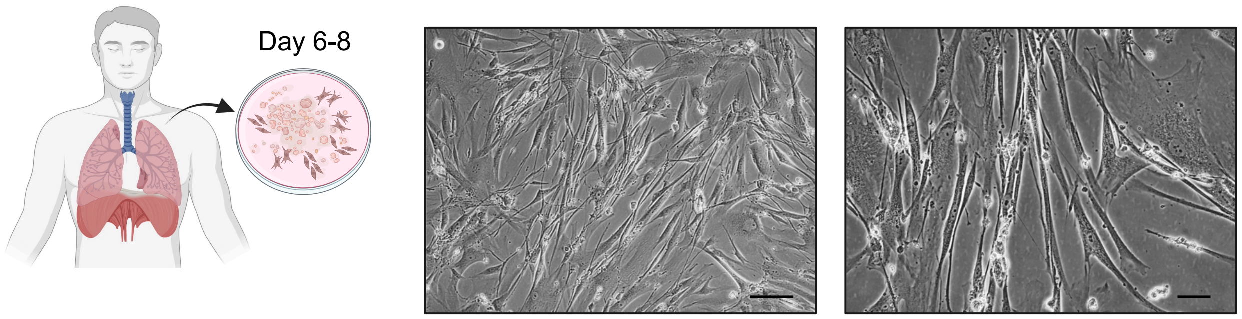

C)

	Sex	Age
1)	M	24
2)	F	23
3)	M	22
4)	M	18
5)	M	19
6)	M	38
7)	M	43
8)	M	28
9)	M	21
10)	M	35
11)	M	21
12)	M	29
13)	M	20
14)	M	23
15)	M	19

D)

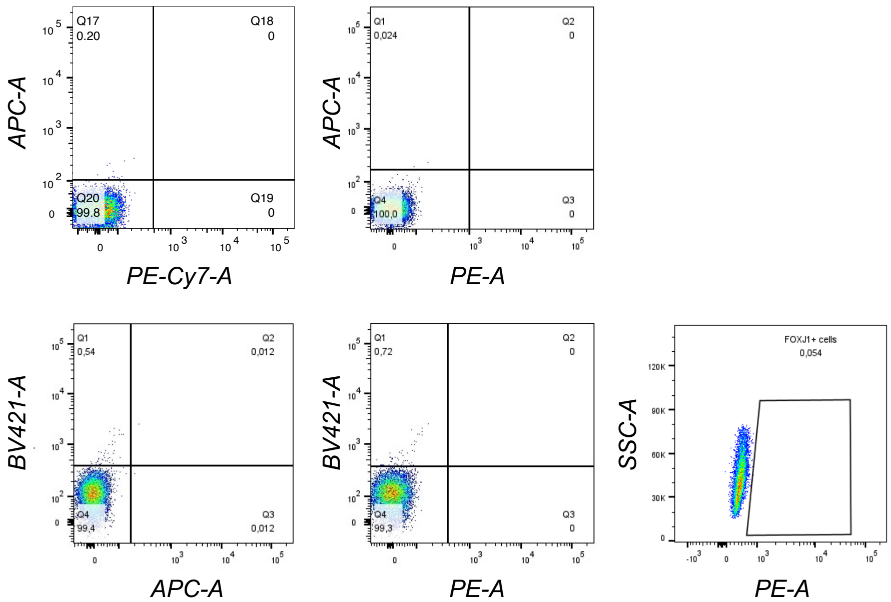

Figure Supplementary 2

A)

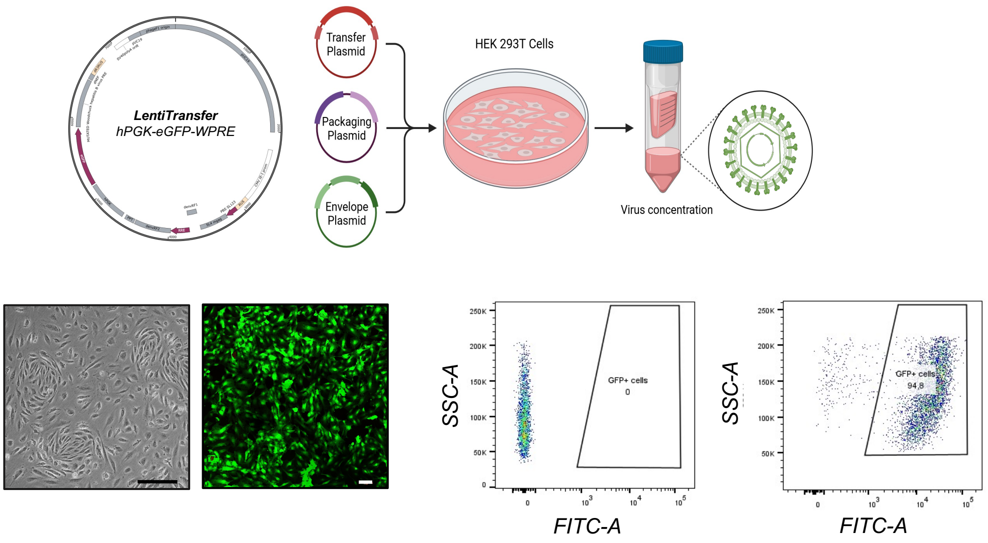

B)

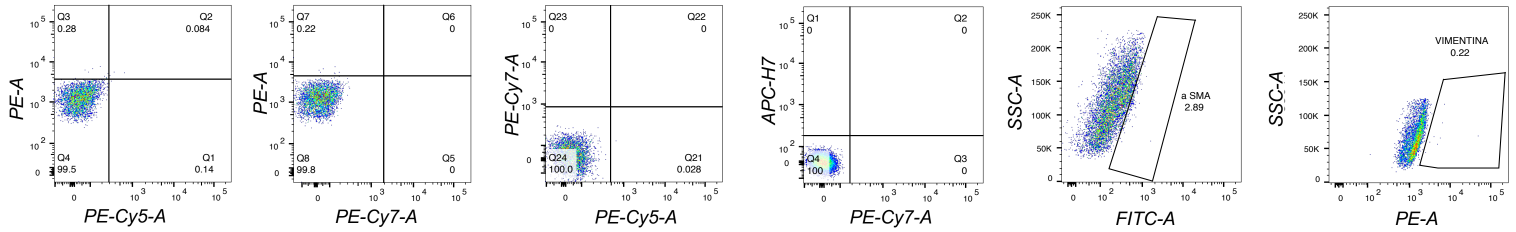

C)

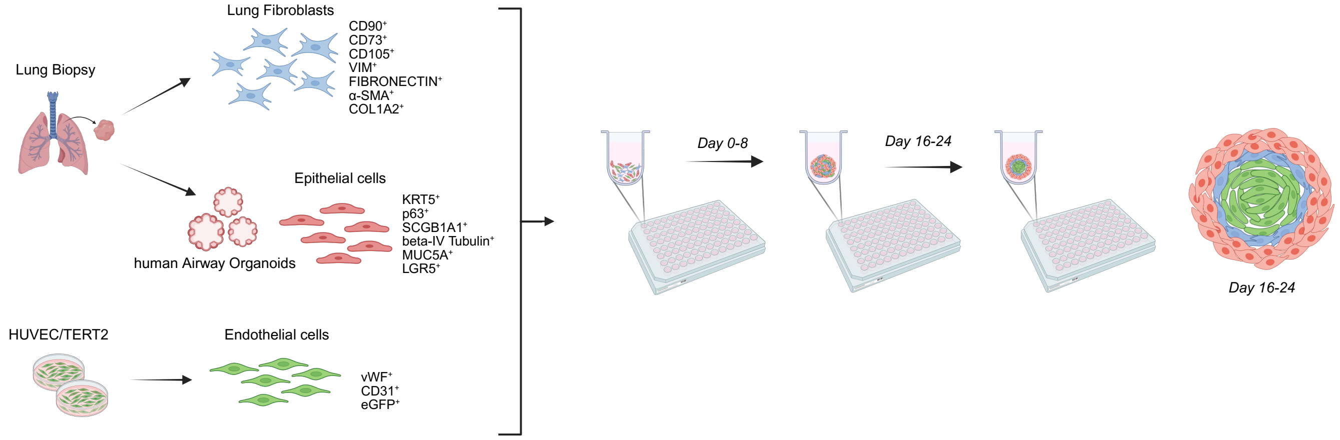

D)

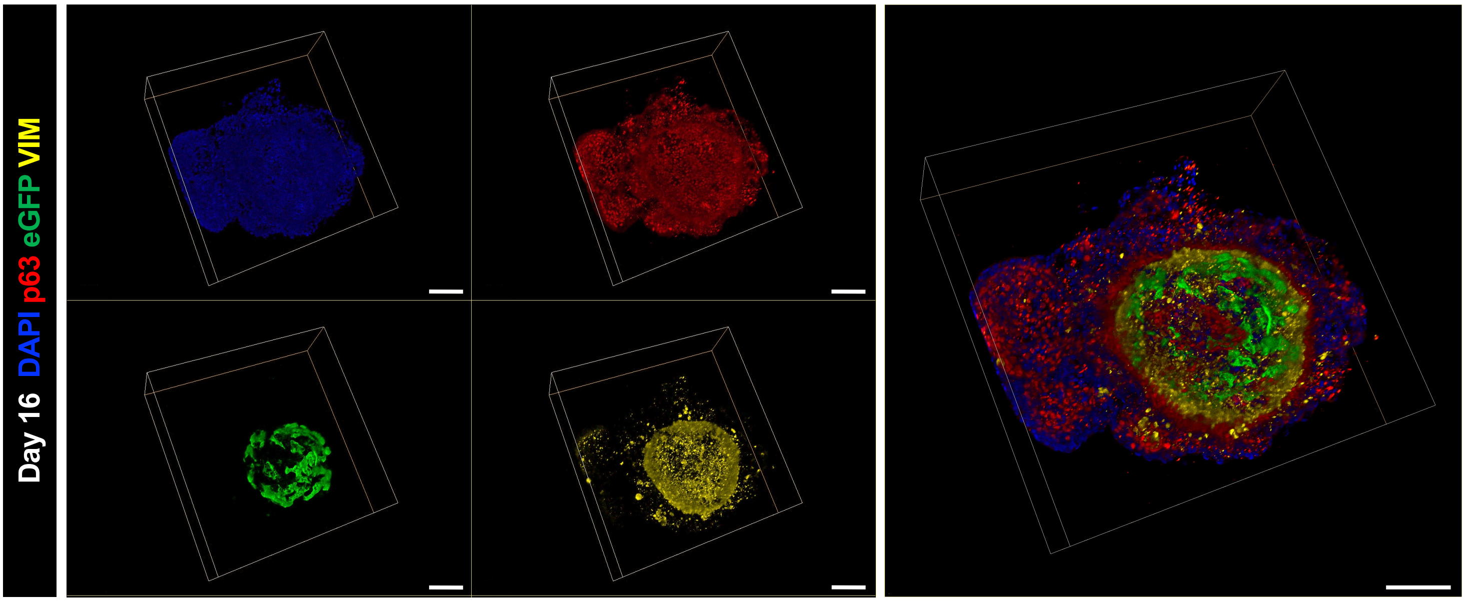

Figure Supplementary 3

A)

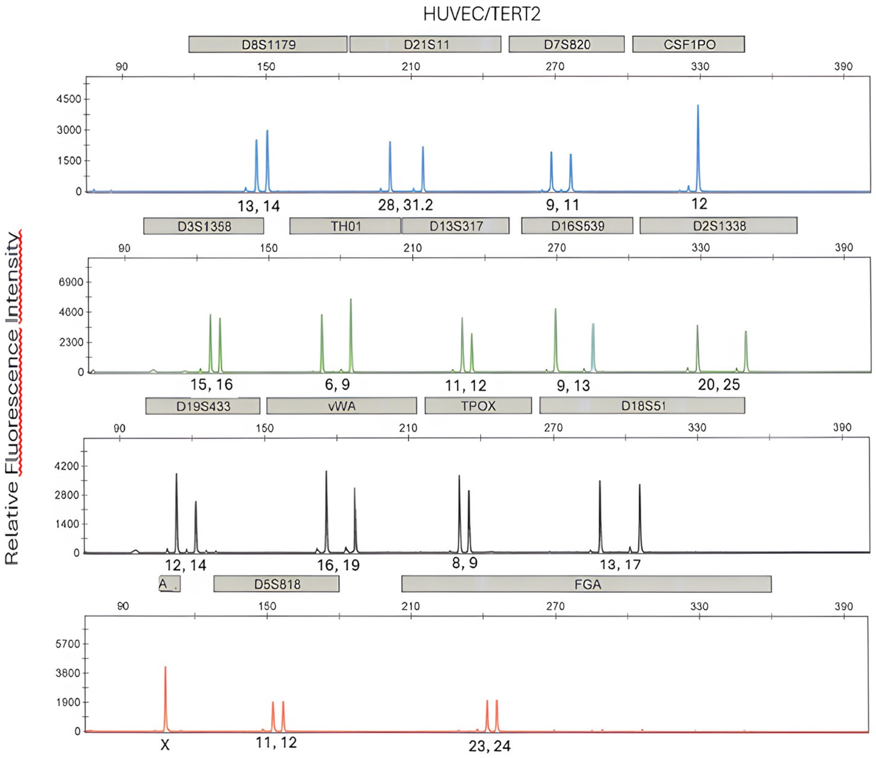

B)

	HUVEC/TERT2	
	Lot# 2021-0084	
AMEL	X	X
D3S1358	15	16
D1S1656	13	16
D2S441	10	14
D10S1248	13	14
D13S317	11	12
Penta E	5	10
D16S539	9	13
D18S51	13	17
D2S1338	20	25
CSF1PO	12	12
Penta D	11	12
TH01	6	9
vWA	16	19
D21S11	28	31,2
D7S820	9	11
D5S818	11	12
TPOX	8	9
D8S1179	13	14
D12S391	20	20
D19S433	12	14
FGA	23	24
D22S1045	11	16

C)

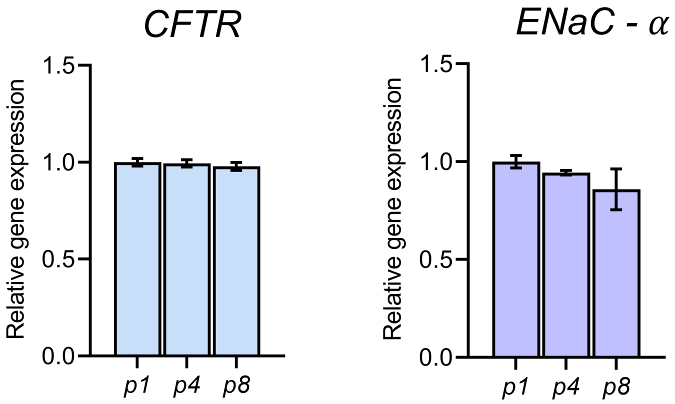

Supplement: Supplementary file 1 — Supplementary figures. [file ijbsv21p6234s1.pdf]
